# An Inflammation‐Related lncRNA Signature for Prognostic Prediction in Colorectal Cancer

**DOI:** 10.1002/cnr2.70043

**Published:** 2024-12-05

**Authors:** Zhenling Zhang, Yingshu Luo, Yuan Liu, Jiangnan Ren, Zhaoxiong Fang, Yanzhi Han

**Affiliations:** ^1^ Department of Gastroenterology, the Fifth Affiliated Hospital Sun Yat‐Sen University Zhuhai China

**Keywords:** colorectal cancer, immune microenvironment, inflammation, lncRNAs, prognosis

## Abstract

**Background:**

Colorectal cancer (CRC) represents a commonly diagnosed malignancy affecting the digestive system. Mounting evidence shows long noncoding RNAs (lncRNAs) contribute to carcinogenesis. However, inflammation‐related lncRNAs (IRLs) regulating CRC are poorly defined.

**Aims:**

The current study aimed to develop an IRL signature for predicting prognosis in CRC and to examine the involved molecular mechanism.

**Methods and Results:**

RNA‐seq findings and patient data were retrieved from The Cancer Genome Atlas (TCGA), and inflammation‐associated genes were obtained from the GeneCards database. IRLs with differential expression were determined with “limma” in R. Using correlation and univariable Cox analyses, prognostic IRLs were identified. The least absolute shrinkage and selection operator (LASSO) algorithm was employed to construct a prognostic model including 13 IRLs. The model's prognostic value was examined by Kaplan–Meier (K‐M) survival curve and receiver operating characteristic (ROC) curve analyses. Furthermore, the association of the signature with the immune profile was assessed. Finally, RT‐qPCR was carried out for verifying the expression of inflammation‐related lncRNAs in nonmalignant and malignant tissue samples. A model containing 13 inflammation‐related lncRNAs was built and utilized to classify cases into two risk groups based on risk score. The signature‐derived risk score had a higher value in predicting survival compared with traditionally used clinicopathological properties in CRC cases. In addition, marked differences were detected in immune cells between the two groups, including CD4^+^ T cells and M2 macrophages. Furthermore, RT‐qPCR confirmed the expression patterns of these 13 lncRNAs were comparable to those of the TCGA‐CRC cohort.

**Conclusion:**

The proposed 13‐IRL signature is a promising biomarker and may help the clinical decision‐making process and improve prognostic evaluation in CRC.

## 1 Introduction

Colorectal carcinoma (CRC) represents the third commonest and second deadliest malignancy worldwide. CRC presents a serious threat to public health [1]. Epidemiological evidence revealed > 1.9 million incident CRC cases in 2020, with 0.9 million deaths [2]. Distant invasion and metastases are the major causes of death in CRC. About 20% of CRC cases show distant metastases, and another 25% eventually develop distant metastases during treatment [3, 4]. At present, no effective molecular markers or predictive models are available to monitor such cases. Therefore, major effectors associated with CRC pathogenesis should be assessed to identify biomarkers and build prognostic models for CRC prevention, diagnosis, and treatment.

The tumor microenvironment (TME) is greatly involved in carcinogenesis and patient outcomes. In addition to malignant cells, the TME encompasses multiple cells such as cancer‐associated fibroblasts, vascular cells, and infiltrating immune cells, which remarkably affect diverse events in cancer cells in a cell non‐autonomous fashion [5]. Inflammation, an important hallmark of cancer, affects the TME via multiple mechanisms, e.g., biosynthesis of proinflammatory factors, angiogenesis, and tissue remodelling. CRC is tightly associated with chronic inflammation, which occurs early in carcinogenesis [6]. Besides cancer cells, local and infiltrated noncancerous cells and the gut microbiota substantially control cell polarization and plasticity in the TME, thereby increasing inflammation‐induced tumorigenesis and metastasis [7]. Currently, predictive models considering inflammation‐associated genes for CRC prognosis are scarce.

Long noncoding RNAs (lncRNAs) are RNAs made of > 200 nucleotides that cannot be translated into proteins [8]. Recent evidence reveals that lncRNAs regulate cancer cell oxidative stress, inflammation, autophagy, and apoptosis [9, 10, 11]. SMAR1 and p53‐regulated lncRNA RP11‐431 M3.1 enhances HIF1A translation via miR‐138 in CRC cells under oxidative stress [12]. LncRNA SP100‐AS1/miR‐622/ATG3 axis contributes to radioresistance and autophagic activity in CRC patients [13]. Telomerase RNA component lncRNA as a potential diagnostic biomarker promotes CRC cellular migration and apoptosis evasion via modulation of β‐catenin protein level [14]. LncRNAs also contribute to inflammatory disorders [15]. The inflammatory TME, resulting from an inflammatory state, is required for and fuels almost all malignancies [16]. Thus, inflammation‐related lncRNAs (IRLs) might be critical in the progression to inflammatory TME during tumorigenesis. For example, LINC00346 silencing suppresses the proliferative, migratory and invasive properties of glioma cells [17]. Recently, researchers have found that the interactions of CCAT1 lncRNA, miR‐185‐3p, and myosin light chain kinase may provide a potential therapeutic option for inflammatory bowel disease and improve early diagnosis and treatment of inflammation‐associated CRC [18]. Similarly, the lncRNA MALAT1 promotes the interaction between BRG1 and NF‐κB pP65 as a scaffold after LPS induction and upregulates the pro‐inflammatory cytokine IL‐6/CXCL8 to enhance hepatocellular carcinoma worsening [19]. However, current studies screening IRLs in CRC are scarce. Consequently, identifying major IRLs with a prognostic value in CRC cases is of high importance.

Here, RNA‐Seq data were retrieved from a colon and rectal adenocarcinoma (COAD‐READ) dataset, and 13 IRLs with differential expression were detected and utilized to develop a prognostic model. Then, how IRLs regulate CRC was further assessed by gene set enrichment analysis (GSEA) and by analysing immune cell infiltration. Moreover, the roles of 13 IRLs in CRC and peritumoral tissues were preliminarily verified.

The current findings may help predict patient prognosis in CRC, providing a reference for individualized therapy.

## 2 Methods

### 2.1 Data Source and Processing

RNA‐Seq data were acquired for 695 COAD‐READ specimens, including 51 noncancerous and 644 tumour specimens, and the respective patient features were retrieved from TCGA. The Bioconductor package in R (version 4.2.1) was employed for analysis.

### 2.2 Identification of Inflammation‐Related lncRNAs

Inflammation‐related genes (IRGs) were retrieved from the GeneCards database (https://www.genecards.org) by entering the keyword “inflammation.” Then, the Spearman correlation coefficient was determined for each IRG and lncRNA profile to obtain IRLs (|*R* ^2^ | > 0.4 and *p* < 0.001).

### 2.3 Differential Expression Analysis

Differentially expressed lncRNAs in TCGA‐COAD‐READ specimens versus noncancerous controls (|log_2_(FC)| > 1 and a false discovery rate [FDR] < 0.05) were detected with “limma” in R.

### 2.4 Establishment of an Inflammation‐Related Prognostic Signature

Genes intersecting between IRLs and differentially expressed lncRNAs (DELs) were screened by Cox univariable analysis using “survival” in R, defining IRLs with potential prognostic value (*p* < 0.001). Next, the least absolute shrinkage and selection operator (LASSO)‐Cox regression analysis was carried out to examine these candidates. Finally, we selected the optimal penalty parameter (λ) associated with the minimum 10‐fold cross‐validation to establish an optimal prognostic model including 13 genes. The inflammation‐related prognostic risk score was calculated as:

$$\text{Risk score}=\sum_{i=1}^{n}\text{Coef}\left(i\right)X\left(i\right)$$

where *x*_*i*_ and coef_*i*_ are the level of a given lncRNA and the respective coefficient, respectively. Based on the median risk score, cases were assigned to the low‐ and high‐risk groups. “survminer” in R was employed to generate Kaplan–Meier curves for overall survival (OS), whose comparison used the log‐rank. Receiver operating characteristic (ROC) curve analysis was carried out to assess the signature for its predictive value using “timeROC” in R.

### 2.5 Functional Enrichment Analysis

Genes with differential expression in the high‐risk group versus low‐risk cases (|log_2_(FC)| > 1 and FDR < 0.05) were obtained with “limma” in R. Then, Gene Ontology (GO) and Kyoto Encyclopaedia of Genes and Genomes (KEGG) analyses were performed for functional annotation using “clusterProfiler” in R (adjusted *p* < 0.05).

### 2.6 Gene Set Enrichment Analysis (GSEA)

Biomolecular differences between low‐ and high‐risk cases were assessed by GSEA considering the KEGG and HALLMARK gene sets from the molecular signature database (https://www.gsea‐msigdb.org/gsea/msigdb) as references, with “clusterProfiler” in R (*p* < 0.05 and FDR < 0.2). Single‐sample GSEA (ssGSEA) was carried out for multiple representative gene groups using “GSVA” in R.

### 2.7 Evaluation of Infiltrated Immune Cells

Infiltrated immune cells in COAD‐READ cases were examined with the ESTIMATE algorithm. Considering the LM22 dataset in CIBERSORT (http://CIBERSORT.stanford.edu/), which includes 22 immune cell subsets, differences in infiltrated immune cells between high‐ and low‐risk cases were assessed with “ggpubr” in R. Immune scores in both patient groups were obtained with “estimate” in R. “ggpubr” in R was also used for plotting immune and stromal scores.

### 2.8 Tissue Sample Collection

All tissue samples were collected from the Gastrointestinal Department of Fifth Affiliated Hospital of Sun Yat‐sen University, which was approved by the Medical Ethics Committee of the hospital. Eleven pairs of samples, including tumour and pericarcinous tissues, were obtained from CRC patients who underwent colonoscopy between October 2022 and June 2023.

### 2.9 RNA Extraction and Quantitative

The real‐time polymerase chain reaction (RT‐qPCR) total tissue RNA was extracted using the FastPure Cell/Tissue Total RNA Isolation Kit V2 (Vazyme, Nanjing, China) according to standard protocols. Then, the obtained RNAs were used for cDNA synthesis with HiScript III RT SuperMix for qPCR (+gDNA wiper) (Vazyme, Nanjing, China). Gene expression was quantified by a Real‐Time PCR Detection System (Bio‐Rad) using ChamQ Universal SYBR qPCR Master Mix (Vazyme, Nanjing, China), and the expression levels were calculated with the 2^−∆∆Ct^ method. GAPDH acted as the internal reference for normalization. The primers designed by Guangzhou IGE Biotechnology (Guangzhou, China) were as indicated in Table S1.

### 2.10 Statistical Analysis

All the statistical analyses were performed with R software (version 4.0.3). The Wilcox test was used to compare the proportion of tumour infiltrating immune cells. Spearman correlation analysis was used to analyse the correlation between IRGs and IRLs. The Kaplan–Meier method was applied to calculate the cumulative survival time and the log‐rank test from the survival package was used to analyse the differences in survival curves. Cox proportional risk regression models were applied for multivariate analyses. *p* < 0.05 means the difference is statistically significant.

## 3 Results

### 3.1 Inflammation‐Associated Differentially Expressed LncRNAs in CRC

**FIGURE 1:**
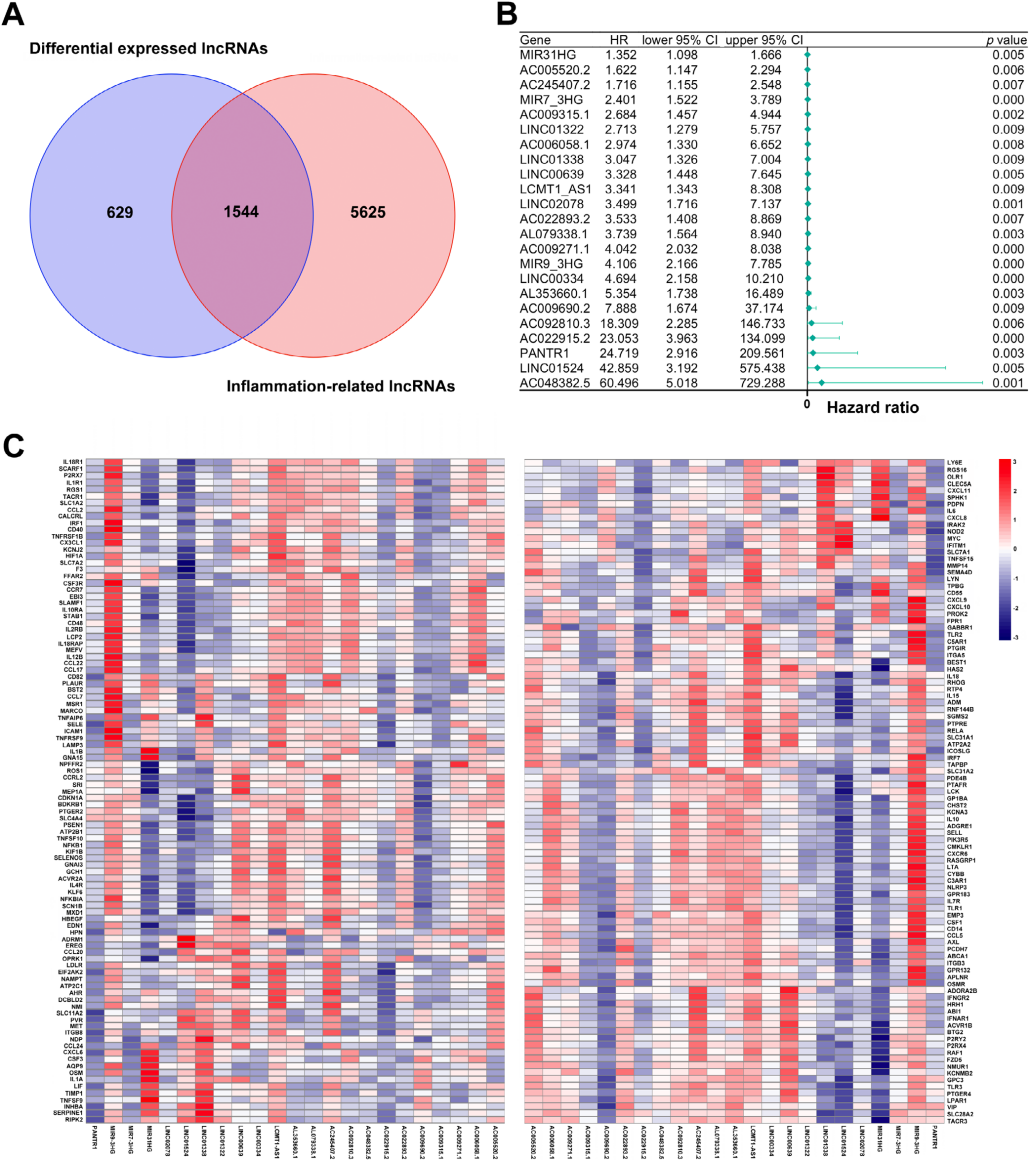
Prognostic analysis of IRDELs. (A) Venn diagram to identify the common lncRNAs of DELs and IRLs. (B) Forest plots showing the results of the Cox univariate regression analysis of approximately 23 prognostic inflammation‐related differentially expressed lncRNAs (IRDELs). (C) The correlation between 23 prognostic IRLs and 196 IRGs in the TCGA‐COAD‐READ cohort. The colour of each unit shows the degree of correlation.

Data for 695 COAD‐READ specimens were retrieved from TCGA, identifying 16 882 lncRNAs and 19 962 mRNAs. To determine genes associated with inflammation, IRGs for the human species were retrieved from GeneCards. Finally, 196 IRGs were obtained and employed to determine IRLs based on Spearman correlation analysis of lncRNAs in TCGA [(|R^2^|) > 0.4 and *p* < 0.001]. As a result, 7169 IRLs were determined. Subsequently, 2173 differentially expressed lncRNAs (DELs) in TCGA‐COAD‐READ specimens between noncancerous and malignant tissues (log_2_|FC| > 1, FDR < 0.05) were identified, comprising 858 upregulated and 1315 downregulated DELs. Finally, 1544 inflammation‐related DELs (IRDELs) were obtained (Figure 1A).

### 3.2 Prognostic Inflammation‐Related DELs

IRDELs were assessed for prognostic value by Cox univariable regression analysis based on OS data of COAD‐READ cases in TCGA. Finally, 23 prognostic IRDELs (PIRDELs) were identified (Figure 1B). The 23 PIRDELs were considered “risk” genes. The associations of the 23 PIRDELs with 196 IRGs are shown in Figure 1C.

### 3.3 Establishment of an IRL Prognostic Model

**FIGURE 2:**
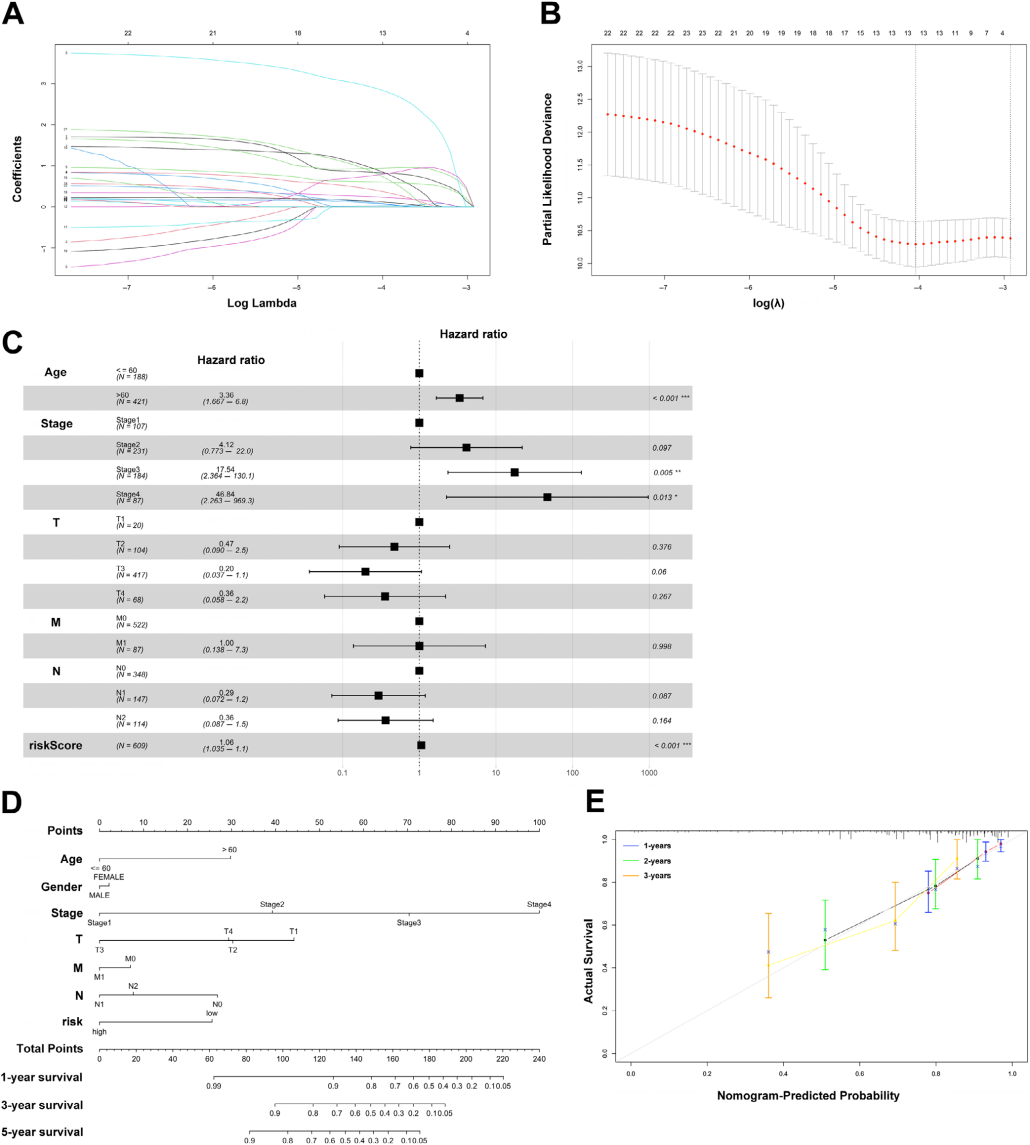
Establishment of a 13‐IRL signature and the analysis of independent prognostic potential. (A, B) cvfit and lambda curves showing the least absolute shrinkage and selection operator (LASSO) regression was performed with the minimum criteria. (C) Results of the multivariate Cox regression analysis regarding OS of the 13‐IRLs signature. (D) Nomogram to predict the 1‐year, 3‐year, and 5‐year overall survival rate of CRC patients. (E) Calibration curve for evaluating the accuracy of the nomogram model. The dashed diagonal line in grey colour represents the ideal nomogram. *p < 0.05, **p < 0.01, ***p < 0.001.

**FIGURE 3:**
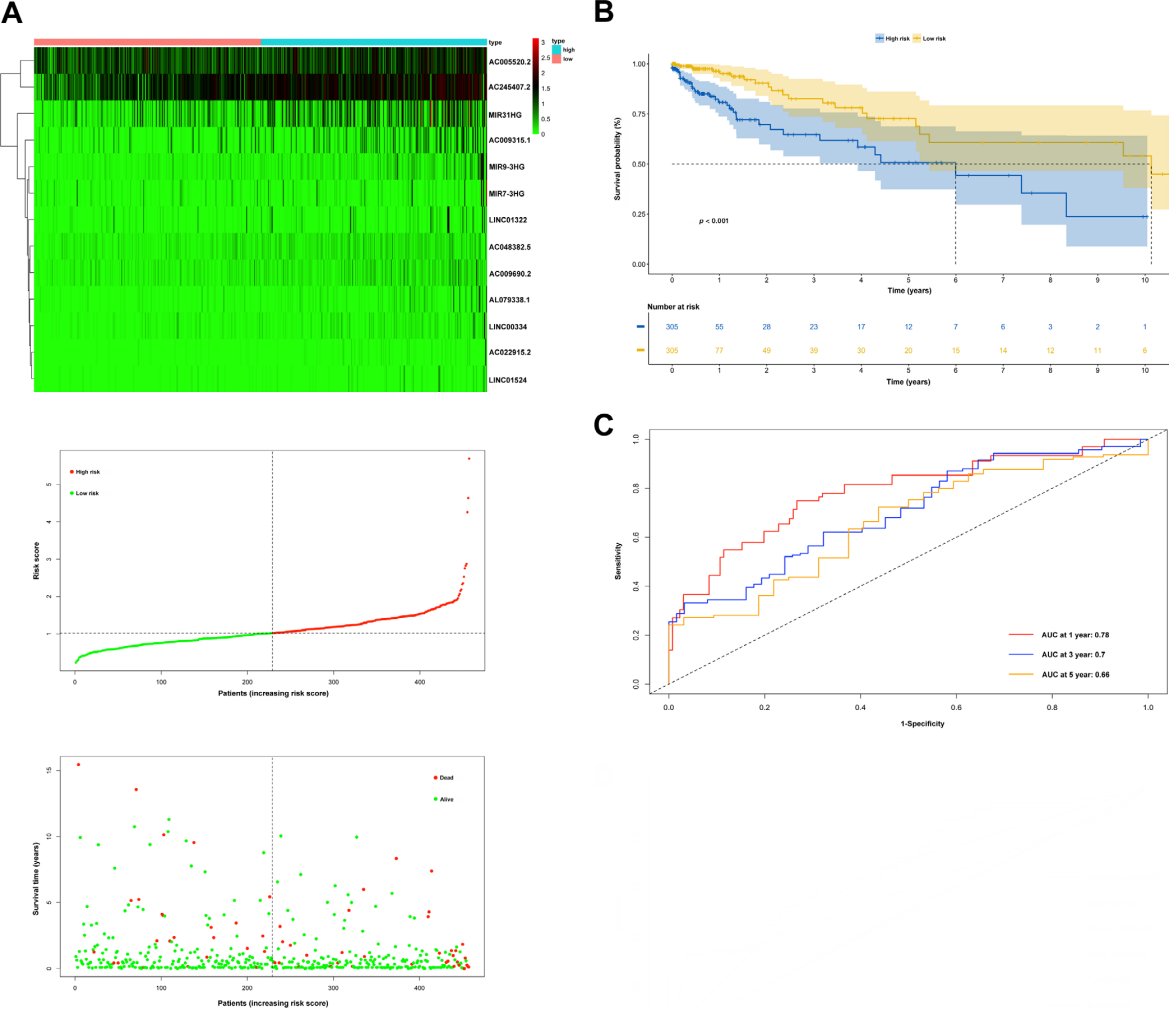
Construction and validation of the IRL signature model. (A) Distribution of the risk scores and the distributions of overall survival status and risk score. (B) The Kaplan–Meier curves for survival status and survival time. (C) Receiver operating characteristic (ROC) curve shows the potential of the prognostic IRLs signature in predicting 1‐, 3‐, and 5‐year overall survival (OS).

Considering only 13 IRLs from the above 23 PIRDELs, a prognostic model was built as described in the experimental section. Figure 2A,B depict cvfit and lambda curves, respectively. A risk score was derived for each COAD‐READ case in TCGA as AC022915.2*2.86410+LINC00334*0.94018+AC048382.5*0.88117+MIR9_3HG *0.83111+LINC01524*0.78149+AC009690.2*0.77836+AL079338.1*0.60326+AC009315.1*0.38909+AC245407.2*0.23709+MIR31HG*0.12376+LINC01322*0.09295+AC005520.2*0.07566+MIR7_3HG*0.02511, where the name of a given lncRNA indicates its expression in TCGA. Cox multivariable regression analysis was carried out to assess the signature for prognostic value, revealing the 13‐IRL risk signature, age, stage 3 and stage 4 independently predicted OS in the COAD‐READ cases of TCGA (*p* < 0.001; Figure 2C). A predictive nomogram assesses the odds of patient survival by summing the scores obtained on the point scale for all factors. The 1‐, 3‐ and 5‐year OS rates were predicted with high accuracy considering the ideal predictive model (Figure 2D,E). Next, the novel 13‐IRL model was examined for prognostic value. Then, cases were assigned to the high‐ and low‐risk groups based on the median risk score. The risk score and OS status distributions indicated a reasonable separation of both risk groups (Figure 3A). In Kaplan–Meier survival analysis, the OS rate was markedly reduced in high‐risk COAD‐READ cases compared with low‐risk counterparts (Figure 3B). Time‐dependent ROC curves revealed areas under the curves (AUCs) above 0.66 at 1‐, 3‐ and 5 years (Figure 3C). High‐risk cases had overtly higher mortality compared with low‐risk cases.

### 3.4 Involved Molecular Functions and Pathways Based on GSEA, GO and KEGG Analyses

**FIGURE 4:**
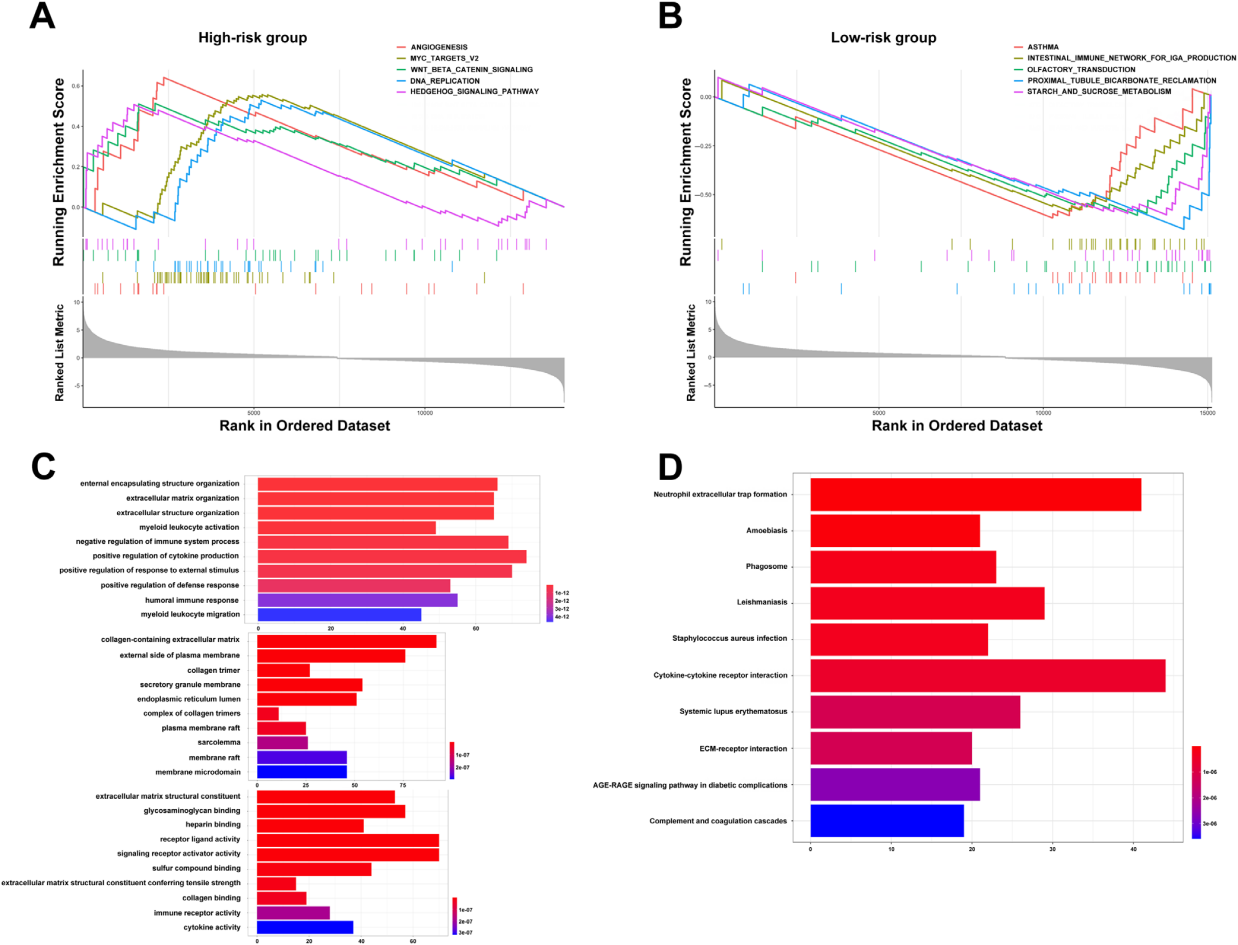
Involved in biological functional and pathway enrichment analysis of high‐risk case and low‐risk cases based on the IRL prognostic signature. (A) GSEA shows significant enrichment of cancer proliferation and metastasis pathways in high‐risk CRC patients. (B) GSEA shows a significant enrichment of intestinal immune and metabolism‐related pathways in low‐risk CRC patients. (C) GO analysis showing many immune‐related biological processes were enriched. (D) KEGG analysis shows many TME pathways were enriched.

GSEA was employed to examine potential differences in biological functions and signalling pathways between the two risk groups based on the 13‐IRL signature. As a result, multiple pathways regulating cancer cell division were enriched in high‐risk cases, including angiogenesis‐associated, Myc targets v2 and DNA replication pathways. Multiple pathways associated with the oncogenesis and development of CRC, for example, Wnt/β‐catenin signalling and Hedgehog signalling, were also retrieved (Figure 4A). Moreover, the intestinal immune network for IgA production as well as starch and sucrose metabolism pathways were enriched in low‐risk cases (Figure 4B). Next, differentially expressed genes (DEGs) between high‐ and low‐risk cases (log_2_|FC| > 1 and FDR < 0.05) were examined by GO and KEGG analyses. KEGG analysis revealed a significant enrichment of multiple immune‐related pathways, e.g., neutrophil extracellular trap formation, phagosome, cytokine‐cytokine receptor interaction, complement and coagulation cascades, and systemic lupus erythematosus (Figure 4D). GO analysis revealed the enriched biological process (BP), molecular function (MF), and cell component (CC) terms (Figure 4C), which corroborated KEGG analysis. Jointly, the above data indicated the risk score derived from the 13‐lncRNA signature was mostly associated with tumour proliferation, metastasis and immunity in CRC.

### 3.5 Assessment of Infiltrated Immune Cells in CRC Cases Considering the Prognostic Signature

**FIGURE 5:**
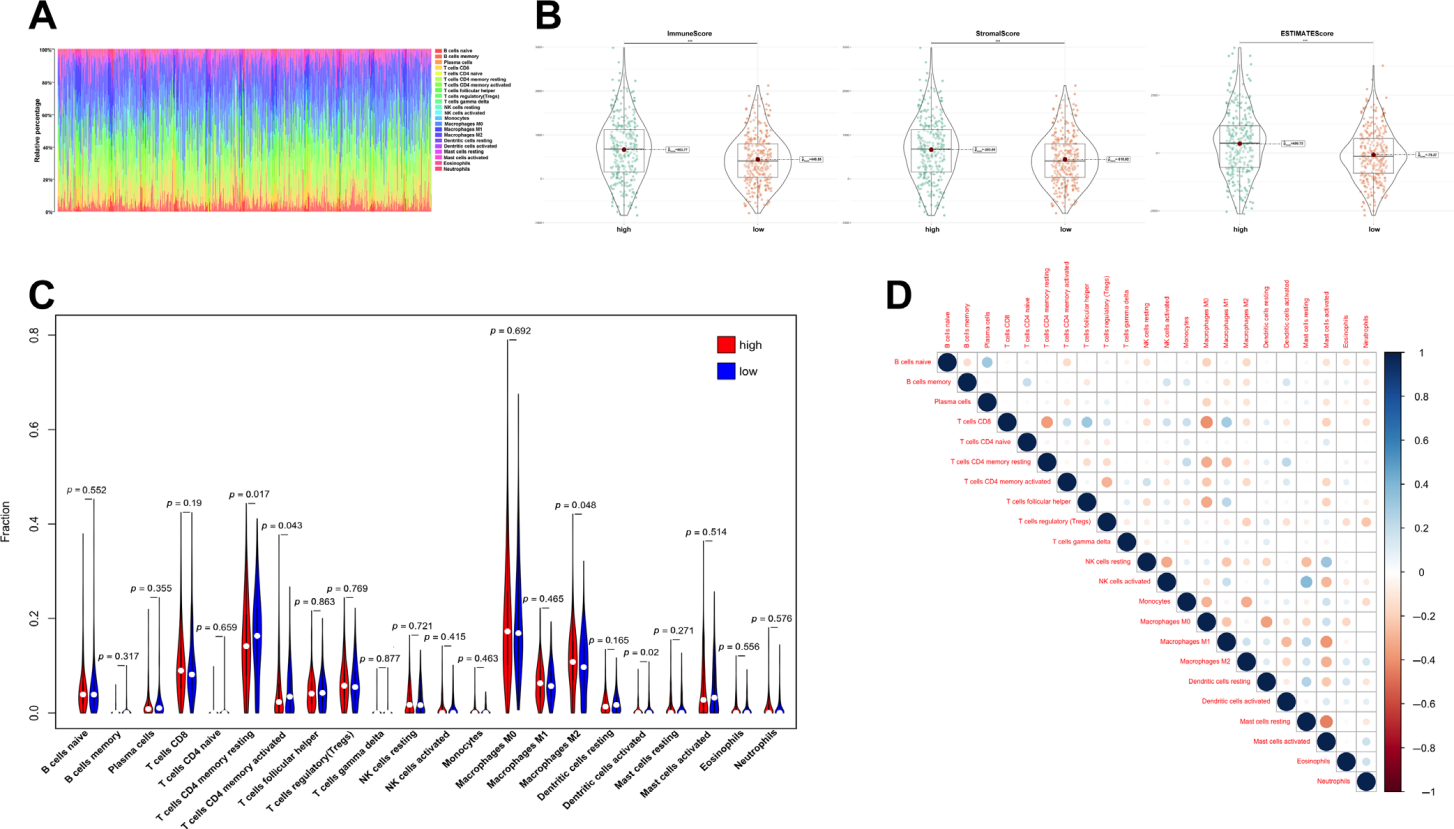
Assessment of infiltrated immune cells in CRC patients. (A) Bar graphs exhibiting the distribution of tumour‐infiltrating immune cells between the high‐risk and low‐risk cases based on the IRLs signature. (B) Stroma, immune, and ESTIMATE scores in the high‐risk and low‐risk cases in CRC patients. (C) Boxplots for the comparison of the 22 immune cells between the high‐risk and low‐risk cases in the CRC patients. (D) Correlation between the infiltrated immune cells. ***p < 0.001.

To further examine the association of the inflammation‐related signature with anti‐tumour immunity in CRC, immune cell infiltration profiles for COAD‐READ cases in TCGA were assessed with CIBERSORT. The proportions of various immune cell types are depicted in Figure 5A. To compare infiltrated immune cells between high‐ and low‐risk cases, stromal (substrate cells detected in the tumour tissue), immune (immune cells infiltrated in the tumour tissue) and ESTIMATE (stromal immune cells) scores were determined. As displayed in Figure 5B, these scores were all significantly elevated in high‐risk cases compared with low‐risk cases (*p* < 0.001). In addition, resting and activated CD4^+^ memory T cells, M2 macrophages, and activated dendritic cells had significantly different levels between the two groups (Figure 5C). Furthermore, many immune cell types showed significant correlations (Figure 5D). Overall, these data suggested that the risk score derived from the 13‐lncRNA signature is associated with infiltrated immune cells in CRC patients.

### 3.6 Validation of IRL Expression

**FIGURE 6:**
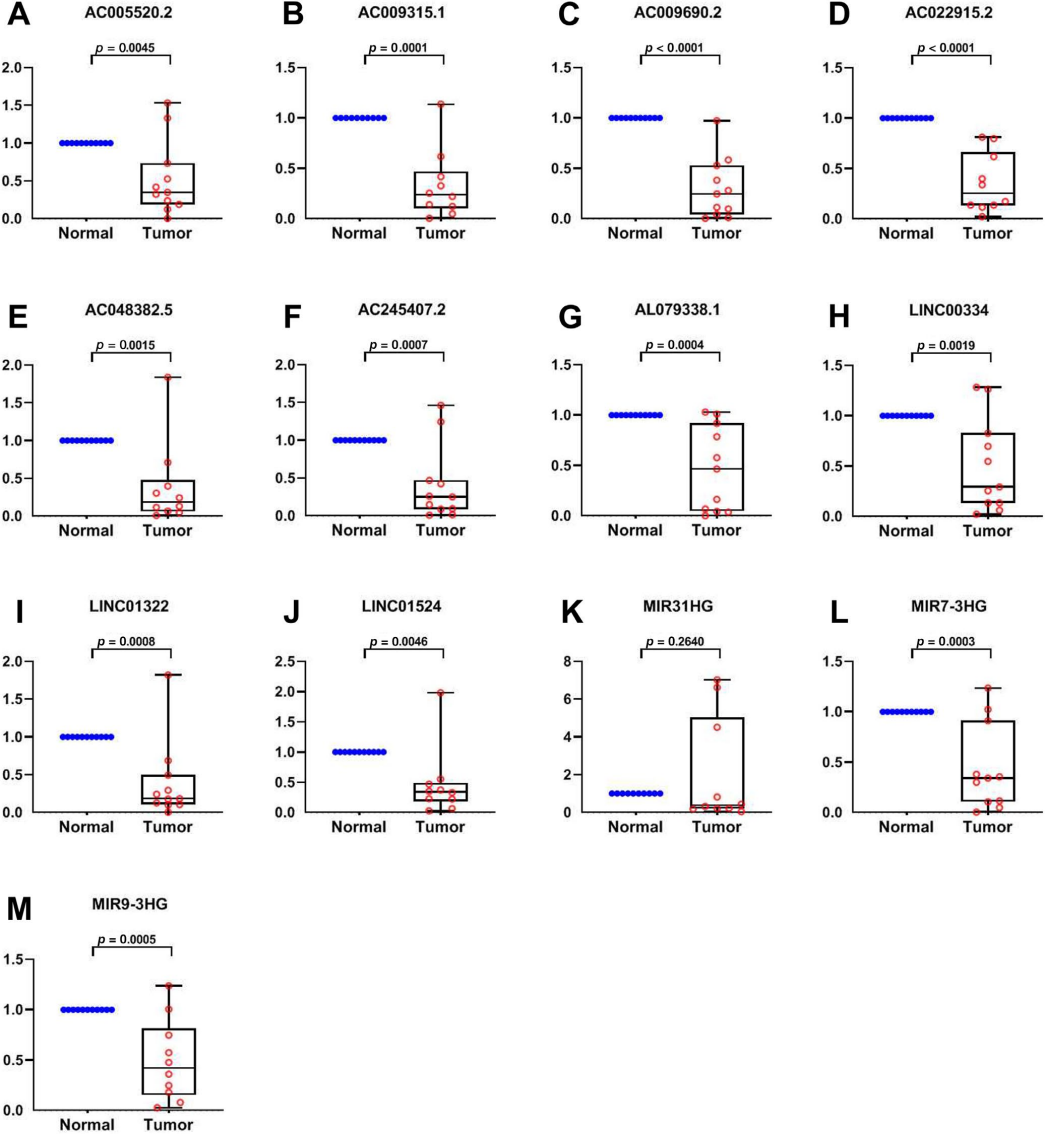
Validation of IRLs expression in tissues. (A‐M) Expression analysis of MIR31HG, MIR9_3HG, LINC00334, MIR7_3HG, AC022915.2, AC048382.5, AC009315.1, AL079338.1, LINC01524, AC005520.2, AC245407.2, AC009690.2 and LINC01322 in 11 pairs of CRC specimens.

The 13 prognostic IRLs were assessed in paired specimens from CRC cases in our hospital. In comparison with peritumoral tissues (normal), MIR31HG showed elevated amounts in tumour tissues (tumour), while MIR9_3HG, LINC00334, MIR7_3HG, AC022915.2, AC048382.5, AC009315.1, AL079338.1, LINC01524, AC005520.2, AC245407.2, AC009690.2 and LINC01322 had lower levels (Figure 6A–M). The above findings are consistent with the TCGA database except for LINC01524 and AC009690.2, further validating the above bioinformatic analysis.

## 4 Discussion

Multiple reports have examined the functions of lncRNAs in inflammatory processes related to cancer [20]. Identifying IRLs is crucial for the development of potential therapeutic targets in cancer. However, the involvement of IRLs in CRC has not been completely elucidated. Here, 196 validated human IRGs in GeneCards were examined, and differentially expressed IRLs were determined. Then, patient prognosis in TCGA was assessed along with the expression of the retrieved IRLs, leading to the identification of 23 prognostic IRLs. An inflammation‐immune‐related lncRNA signature was built considering the retrieved lncRNAs. Specifically, this signature had an enhanced capability of predicting patient prognosis in CRC compared with the routine TNM stage. Multiple adverse events, including venous invasion and lymphatic metastasis, may also be predicted using the novel risk score. Next, cases were assigned to the high‐ and low‐risk groups according to median risk score, which showed significant differences in TME and immunotherapy response.

To further examine the signature's value in CRC, GSEA was carried out. As shown above, the angiogenesis pathway ranked high in high‐risk cases, and angiogenesis is important in the pathogenesis of cancer [21]. Cancer proliferation, apoptosis, growth and metastasis pathways such as Myc targets v2 [22], Wnt/β‐catenin [23] and Hedgehog [24] were also enriched. Chronic inflammation increases cancer risk and promotes tumour growth by enhancing tumorigenic and metastatic mechanisms [25]. Mounting evidence shows tumorigenesis might be triggered by an inflammatory TME, particularly in CRC. In the present work, immune‐related hallmark terms were enriched, including the intestinal immune network for IgA production. Therefore, it can be reasonably assumed that tumour immunity is highly associated with inflammation in CRC. Altered metabolism and abnormal biochemical processes are involved in inflammation [26]. Multiple metabolic pathways, for example, carbohydrate metabolism, were also enriched. Genes regulating starch and sucrose metabolism were recently shown to be associated with decreased prognosis in CRC cases [27]. KEGG and GO (BP, MF and CC) analyses yielded findings relatively similar to GSEA data. These findings suggest immune‐related pathways might be crucial for inflammation‐related lncRNAs controlling the immune microenvironment and tumour growth in CRC.

Previous reports have also suggested inflammation is closely associated with tumour immunity [28]. CD4‐positive T cells and tumoricidal myeloid cells induce remote inflammatory cell death, which indirectly eliminates interferon‐unresponsive and MHC‐deficient tumours [29]. TME‐derived signals induce peripheral blood monocytes to differentiate into tumour‐associated macrophages (TAMs). A meta‐analysis suggested that elevated macrophage infiltration in solid tumours generally indicates poor prognosis [30]. Since some individuals do not substantially benefit from treatment with new immune‐checkpoint inhibitors, TAMs might partly explain the treatment resistance. Indeed, TAMs are highly plastic and traditionally divided into the anticancer M1 and pro‐tumorigenic M2 phenotypes. While inflammation initially fosters an M1‐like phenotype, continued inflammatory stimulation progressively leads to an immunosuppressive, M2‐like phenotype [31]. However, inflammation and infiltrated immune cells have no known associations with CRC. Since multiple immune pathways were retrieved by GSEA, the proportions of various tumour‐infiltrating immune cells in CRC cases in TCGA were determined with CIBERSORT. Not surprisingly, high‐risk cases had markedly elevated immune, stromal and ESTIMATE scores in comparison with low‐risk cases. It was previously shown elevated immune and stromal scores, and enhanced macrophage infiltration are linked to poor prognosis, corroborating the current findings [32]. Additionally, high‐risk cases also displayed relatively reduced amounts of immune cells, including dendritic cells. CD4^+^ T cell responses regulate the immune cycle in tumours, and both are important in patient outcomes [33]. As shown above, CD4^+^ T cells were remarkably decreased in high‐risk cases, indicating CD4^+^ cell functions in CRC patients may be relatively suppressed.

Additionally, we evaluated the 13 prognostic IRDELs in the novel signature for their expression. Besides LINC01524 and AC009690.2, the expression trends essentially corroborated the bioinformatic analysis. MIR31HG functions as an oncogenic gene in CRC [34], while LINC01524 is associated with overall survival in gastric cancer with Hp infection [35].

This study had limitations. First, no external validation was performed because lncRNA profiles were not available in other databases besides TCGA. Secondly, although the 13 lncRNAs were assessed by RT‐qPCR in 11 paired clinical specimens, the sample size was relatively low, and a large sample analysis is required to confirm our findings. Finally, how the 13 lncRNAs regulate inflammation deserves further attention.

In conclusion, a promising prognostic model with 13 IRLs was developed, showing a higher predictive value than routine clinicopathologic features, with a relative ease of testing. The association of the novel risk model with the immune profile was preliminarily examined. This work provides crucial insights into the development of prognostic tools for CRC cases and might improve treatment decision‐making in the clinic.

## Author Contributions

**Zhenling Zhang:** formal analysis, funding acquisition, writing – original draft. **Yingshu Luo:** software, data curation. **Yuan Liu:** methodology, resources. **Jiangnan Ren:** data curation, resources. **Zhaoxiong Fang:** resources. **Yanzhi Han:** conceptualization, writing – review and editing, project administration, supervision.

## Ethics Statement

The studies involving humans were strictly reviewed and approved by the Medical Ethics Committee of the Fifth Affiliated Hospital of Sun Yat‐sen University (approval No. K33‐1, ethical review 2021, the Fifth Affiliated Hospital of Sun Yat‐sen University). The studies involving patient tissue samples obtained written informed consent.

## Conflicts of Interest

The authors declare no conflicts of interest.

## Supporting information


Table S1.

## Data Availability

The datasets analysed during the current study are available from The Cancer Genome Atlas (TCGA) database. Other datasets used and/or analysed during the current study are available from the corresponding author on reasonable request.
